# Effects of rhythm control on left atrial structure remodeling in atrial fibrillation and heart failure with preserved ejection fraction

**DOI:** 10.3389/fcvm.2023.1265269

**Published:** 2023-09-25

**Authors:** Lin Bai, Yuxi Sun, Jiping Si, Zijie Ding, Xinxin Zhang, Yanli Zhang, Yunlong Xia, Ying Liu

**Affiliations:** ^1^ Heart Failure and Structural Cardiology Ward, The First Affiliated Hospital of Dalian Medical University, Dalian, China; ^2^ Department of Cardiology, West China Hospital, Sichuan University, Chengdu, China

**Keywords:** heart failure with preserved ejection fraction, rhythm control, atrial fibrillation, left atrial reverse remodeling, rate control

## Abstract

**Background:**

The benefits of rhythm control for atrial fibrillation (AF) in heart failure with preserved ejection fraction (HFpEF) have not been conclusively determined. We assessed the effects of rhythm control on left atrial (LA) structure remodeling and prognosis in patients with AF and HFpEF.

**Methods:**

This was a retrospective, real-world, observational study involving patients diagnosed with AF and HFpEF. The cohort was divided into rhythm-control and rate-control groups depending on their treatment strategies. The primary outcomes were all-cause mortality, rehospitalization for any cause, HF-related rehospitalization, and stroke. Differences in follow-up LA structure parameters were also analyzed.

**Results:**

Compared to the rate-control group, patients in the rhythm-control group had a lower risk of HF-related rehospitalization even after adjusting for potential confounders (adjusted HR 0.605, 95% CI 0.413–0.887, *p* = 0.010). Moreover, rhythm-control therapy led to marked reductions in LA echocardiographic indicators and a higher proportion of LA reverse remodeling (LARR).

**Conclusions:**

Rhythm-control therapy reverses LA structure remodeling and is associated with improved clinical outcomes; therefore, it is an optimal treatment approach for AF in HFpEF patients.

## Introduction

Globally, approximately 64.3 million people are suffering from heart failure (HF), among whom, heart failure with preserved ejection fraction (HFpEF) accounts for about 36% of the total HF spectrum (1). Atrial fibrillation (AF) is one of the most common complications in HFpEF patients. The Olmsted County cohort study revealed that approximately two-thirds of HFpEF cases had AF during the disease process (2). In another study, AF patients were 4.8 times more likely to develop HFpEF than those without AF (3). These two diseases often share common risk factors, including advanced age, female sex, anemia, and comorbidities such as hypertension, obesity, chronic renal insufficiency, atherosclerosis, and sleep apnea. Additionally, AF and HFpEF interact and promote their progress through cardiac structure and electrophysiology remodeling, neurohumoral activation, inflammation, and other pathogenic mechanisms, resulting in a vicious cycle (4).

The latest therapeutic guidelines recommend that rhythm control therapy, particularly catheter ablation (CA), is a potentially effective strategy for treating symptomatic AF and HF (5, 6). There is sufficient evidence that patients with heart failure with reduced ejection failure (HFrEF) patients and AF who were subjected to catheter ablation (CA) to restore sinus rhythm (SR) exhibited better prognostic outcomes compared to those treated with rate-control therapy (7–12). However, little attention has been paid to HFpEF, and a limited number of studies have been conducted to assess the efficacy of rhythm control therapy (CA, electrocardioversion, or surgical ablation) for HFpEF and AF. According to the available evidence, HFpEF populations may benefit from rhythm control therapies. Thus, to verify it, we investigated the effect of rhythm-control therapy on prognosis and left atrial remodeling in patients with HFpEF and AF.

## Material and methods

### Study population

This was a single-center retrospective cohort study. Consecutive AF and HFpEF patients hospitalized at The First Affiliated Hospital of Dalian Medical University between January 2011 and December 2020 were enrolled. In this study, adult patients received rhythm control (CA, electrocardioversion, or surgical ablation) or rate control therapies (β-blockers or digoxin). The exclusion criteria were end-stage renal failure, absence of electrocardiogram (ECG) findings, and lack of follow-up data. This study was approved by the institutional review board of Dalian Medical University, Liaoning, China, and was conducted under the declaration of Helsinki. All participants involved provided informed consent.

### Grouping

**Figure 1:**
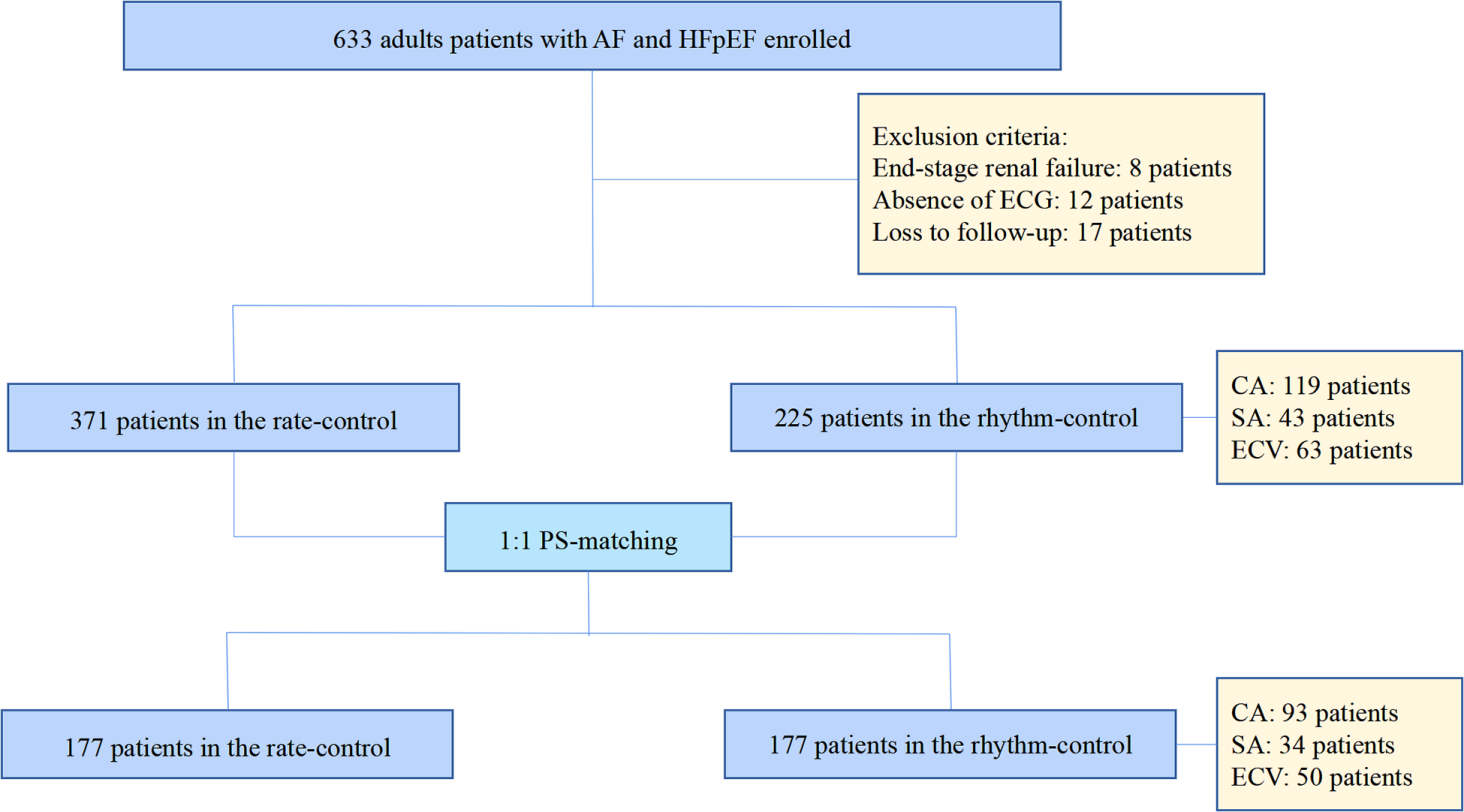
The flow chart of the study population.

The cohort was classified into rhythm and rate control groups depending on their treatment strategies. Patients treated with CA, electrocardioversion (ECV) (100J or 150J), or surgical ablation (SA) (COX Maze) were identified as the rhythm control group. CA is a standard procedure. Following reconstruction of the 3D model of the left atrium (LA) using the Carto 3 (Biosense Webster, USA) 3D mapping system, a catheter procedure was performed to isolate the pulmonary vein. After pulmonary vein isolation, other ablation strategies, such as the LA roof line, mitral isthmus line, tricuspid isthmus line, and BOX ablation, among others, were completed at physicians’ discretion. All patients in the rhythm group received standard anticoagulation (warfarin or novel oral anticoagulants) for at least three weeks and underwent transesophageal echocardiography to rule out the risk of cardiac thrombosis before the rhythm treatment. Postoperatively, patients were administered amiodarone (200 mg 3 times a day for the first week, 200 mg twice daily for the second week, and 200 mg once daily for the third week) to maintain sinus rhythms. By contrast, patients who had been administered with β-blockers or digoxin (without antiarrhythmic medications) to control their ventricular rates were assigned to the rate control group, with the target heart rate <80 bpm. at rest or <110 bpm. during moderate exercise. To reduce selection bias and potential confounding factors, a 1:1 optimal propensity-score (PS) matching with a 0.02 caliper and no replacement was conducted based on age, NYHA class IV, coronary artery disease, hypertension, diabetes, and warfarin. The flow chart of the study population is shown in Figure 1.

### Definitions

The diagnosis of HFpEF and AF is determined in accordance with the guideline of the European Society of Cardiology (6). The diagnostic criteria for HFpEF included: (i) typical signs and symptoms of HF; (ii) LVEF ≥50%; and (iii) evidence of spontaneous or provokable increased LV filling pressures (elevated brain natriuretic peptide, non-invasive and invasive hemodynamic measurements). Clinically, AF is defined as ≥30 s recorded in a standard 12-lead ECG or single-lead ECG, with no identifiable repetitive *P* waves and irregular RR intervals when atrioventricular conduction was not impaired.

### Clinical data

Demographics, the New York Heart Association (NYHA) functional class, co-morbidities, laboratory tests, and information on drug therapies were collected. Some valuable echocardiographic parameters listed as follows were also recorded. Mitral Doppler early velocity/mitral annular early velocity (E/e'), the ratio of peak velocity of mitral inflow during early diastole (E), and over the average septal and lateral mitral annular early diastolic peak velocities (e'), reflects left ventricular (LV) stiffness and fibrosis. Left atrial volume index (LAVI), an essential indicator of chronic LA remodeling, is measured at end-systole and indexed to body surface area. Left ventricular mass index (LVMI) and relative wall thickness (RWT) are important markers of global LV remodeling or hypertrophy. LVMI is calculated using the Cube formula and indexed to body surface area. RWT is twice the LV posterior wall thickness divided by the LV internal diameter at end-diastole. Tricuspid regurgitation (TR) peak velocity is considered to be an indirect marker of LV diastolic dysfunctions (13). Left atrial reverse remodeling (LARR), a vital index of LA structure reverse remodeling, is defined by a ≥15% reduction in LA end-systolic volume (LAESV) (14).

### Endpoints on follow-up

The primary clinical outcomes in this study were all-cause mortality, rehospitalization for any cause, HF-related readmission, and stroke. Differences in follow-up LA structure parameters were calculated and compared. The time interval between the two echocardiogram examinations was at least 3 months. Postoperatively, all participants received at least three outpatient visits after ablation (3, 6, and 12 months within 1 year after surgery and once every 6 months after 1 year). During every patient visit or when they felt uncomfortable, an ECG or Holter examination was performed to monitor atrial arrhythmia. A telephone follow-up was made once they did not attend their scheduled appointments. The deadline for follow-up was December 30, 2021.

### Statistical analysis

Normally or non-normally distributed continuous variables were expressed as mean ± standard deviation or median (interquartile range). They were analyzed using an independent-sample *t*-test or Mann-Whitney's *U*-test. Categorical variables were presented as percentages (%), and differences were assessed using a chi-square test. Cumulative incidences of prespecified endpoints were calculated through the Kaplan-Meier curve, with log-rank tests comparing the differences. Cox proportional hazard models were used to compare the risks of adverse events after adjustments, with hazard ratios (HR) and 95% confidence intervals (95% CI) presented. All factors with *p* < 0.05 in the univariate Cox analysis were entered into the multivariate model. *p* < 0.05 was the threshold for statistical significance. The Statistical Package for Social Sciences (SPSS) version 26.0 was used for analysis.

## Results

### Baseline characteristics

A total of 596 patients with HFpEF and AF were initially enrolled. Of these, 225 cases were assigned to the rhythm-control group and 371 to the rate-control group. A 1:1 optimal PS matching with a 0.02 caliper and no replacement was conducted to reduce selection bias and potential confounding factors, yielding 177 successfully matched pairs from the entire cohort.

In the pre-matched cohort, patients who received rate-control therapies were significantly older (72.09 ± 0.52 vs. 64.78 ± 0.66, *p* < 0.001), more likely to suffer from NYHA class IV (13.2% vs. 7.6%, *p* = 0.033), coronary artery disease (24.0% vs. 8.0%, *p* < 0.001), hypertension (65.0% vs. 52.0%, *p* = 0.002), diabetes (29.9% vs. 19.6%, *p* = 0.005), more often took β-blocker (88.1% vs. 45.8%, *p* < 0.001), digoxin (27.8% vs. 3.1%, *p* < 0.001). In contrast, those in the rhythm-control group exhibited higher frequencies of valvular disease (44.0% vs. 30.5%, *p* = 0.001), had higher hemoglobin levels (137.36 ± 1.23 vs. 130.74 ± 1.07, *p* < 0.001), and took more warfarin (65.8% vs. 39.4%, *p* < 0.001). After matching, most variables were comparable among the two groups. In the PS-matched rhythm-control subgroup, the number of cases treated with CA, ECV, and SA were 93, 50, and 34, respectively. The baseline characteristics before and after PS-matching are shown in Table 1.

**Table 1:** Baseline characteristics between rhythm and rate-control groups before and after PS-matching.

	Rhythm-control group	Rate-control group	*p* value	Rhythm-control group (PS matching)	Rate-control group (PS matching)	*p* value
	(*N* = 225)	(*N* = 371)		(*N* = 177)	(*N* = 177)	
Age (y)	64.78 ± 0.66	72.09 ± 0.52	<0.001	66.67 ± 0.71	66.72 ± 0.66	0.958
Male (%)	100 (44.4)	156 (42.0)	0.567	74 (41.8)	74 (41.8)	1.000
NYHA class I–II (%)	72 (32.0)	101 (27.2)	0.213	55 (31.1)	55 (31.1)	1.000
NYHA class III (%)	136 (60.4)	221 (59.6)	0.833	109 (61.6)	110 (62.1)	0.913
NYHA class IV (%)	17 (7.6)	49 (13.2)	0.033	13 (7.3)	12 (6.8)	0.836
Heart rate (bpm)	85 (70, 111)	83 (60, 103)	0.332	85 (70.5, 111.5)	86 (71, 104.5)	0.392
BMI (kg/m^2^)	27.73 ± 2.15	24.91 ± 0.43	0.120	24.48 ± 2.66	24.45 ± 0.65	0.142
Coronary artery disease (%)	18 (8.0)	89 (24.0)	<0.001	18 (10.2)	17 (9.6)	0.859
Valvular disease (%)	99 (44.0)	113 (30.5)	0.001	74 (41.8)	68 (38.4)	0.515
Dilated cardiomyopathy (%)	7 (3.1)	5 (1.3)	0.137	15 (1.1)	16 (2.3)	0.410
Hypertrophic cardiomyopathy (%)	15 (6.7)	27 (7.3)	0.778	15 (8.5)	27 (9.0)	0.851
Hypertension (%)	117 (52.0)	241 (65.0)	0.002	97 (54.8)	102 (57.6)	0.592
Diabetes (%)	44 (19.6)	111 (29.9)	0.005	34 (19.2)	42 (23.7)	0.300
Atrial fibrillation type						
Persistent atrial fibrillation	180 (80.0)	294 (79.2)	0.825	147 (83.1)	141 (79.7)	0.413
Paroxysmal atrial fibrillation	45 (20.0)	77 (20.8)	0.825	30 (16.9)	36 (20.3)	0.413
Drugs						
β-blocker (%)	103 (45.8)	327 (88.1)	<0.001	79 (44.6)	159 (89.8)	<0.001
Digoxin (%)	7 (3.1)	103 (27.8)	<0.001	4 (2.3)	52 (29.4)	<0.001
ACEI or ARB or ARNI (%)	75 (33.3)	143 (38.5)	0.200	58 (32.8)	65 (36.7)	0.435
Aldosterone antagonists (%)	109 (48.4)	186 (50.1)	0.689	85 (48.0)	89 (50.3)	0.671
Diuretic (%)	114 (50.7)	215 (58)	0.083	91 (51.4)	100 (56.5)	0.337
Warfarin (%)	156 (65.8)	176 (39.4)	<0.001	116 (65.6)	111 (62.7)	0.580
Direct oral anticoagulants (%)	64 (28.4)	118 (31.8)	0.388	56 (31.6)	47 (26.6)	0.292
Laboratory test						
HB (g/L)	137.36 ± 1.23	130.74 ± 1.07	<0.001	135.07 ± 1.27	134.76 ± 1.47	0.873
BNP (pg/ml)	233.66 (124.77, 417.95)	264.10 (134.99, 506.92)	0.114	253.49 (138.08, 435.78)	267.32 (129.76, 494.34)	0.928
hs-cTnI (μg/L)	0.36 ± 0.131	0.18 ± 0.055	0.195	0.23 ± 0.11	0.33 ± 0.14	0.587
Cre (μmoI/L)	69 (59, 82)	72 (60, 88)	0.062	68,5 (59, 81.75)	72 (60, 85.5)	0.206
Uric acid (μmoI/L)	400.64 ± 8.06	386.79 ± 6.22	0.175	401.79 ± 9.11	396.45 ± 9.08	0.679
Serum potassium (mmol/L)	4.04 ± 0.02	4.02 ± 0.02	0.549	4.03 ± 0.03	3.99 ± 0.03	0.390
Echocardiographic parameters						
LVEF (%)	56.74 ± 0.17	56.29 ± 0.15	0.053	56.86 ± 0.19	56.38 ± 0.19	0.079
LAD (mm)	45.02 ± 0.60	45.37 ± 0.44	0.638	45.88 ± 0.73	46.65 ± 0.69	0.442
LASID (mm)	47.90 ± 0.69	48.20 ± 0.39	0.685	47.90 ± 0.83	48.20 ± 0.60	0.986
LATD (mm)	64.11 ± 0.82	65.03 ± 0.63	0.369	66.49 ± 0.97	66.72 ± 1.03	0.387
LAV (ml)	63.80 (49.74, 88.00)	68.61 (53.13, 92.73)	0.092	67.54 (51.88, 92.12)	68.61 (57.40, 96.28)	0.149
LAVI (ml/m^2^)	40.42 (30.45, 56.00)	39.73 (30.70, 53.01)	0.773	40.53 (31.43, 59.23)	42.66 (33.25, 57.89)	0.818
IVS (mm)	10.00 (9.00, 11.00)	10.00 (10.00, 12.00)	0.905	11.00 (10.00, 12.00)	10.00 (9.00, 12.00)	0.267
LVPWT (mm)	10.35 ± 0.24	10.16 ± 0.07	0.364	10.52 ± 0.30	10.19 ± 0.10	0.305
LVEDD (mm)	47.32 ± 0.40	47.37 ± 0.32	0.512	47.53 ± 0.45	47.69 ± 0.45	0.791
LVMI (g/m^2^)	97.41 (83.22, 118.63)	97.19 (85.42, 114.27)	0.938	100.94 (83.79, 123.28)	99.37 (85.91, 114.64)	0.757
RWT (mm)	0.44 ± 0.009	0.44 ± 0.003	0.509	0.45 ± 0.01	0.43 ± 0.01	0.312
TRV (m/s)	2.70 (2.40, 3.10)	2.80 (2.50, 3.20)	0.105	2.70 (2.45, 3.20)	2.80 (2.50, 3.30)	0.259
E/e’	10.85 (7.52, 15)	11 (8.6, 14.28)	0.309	11.00 (7.78, 15)	11 (8.3, 15.00)	0.651

BNP, brain natriuretic peptide; IVS, interventricular septal thickness; LAD, left atrial diameter; LASID, left atrium superior-inferior diameter; LATD, left atrium transverse diameter; LAV, left atrial volume; LAVI, left atrial volume index; LVEDD, left ventricular end-diastolic diameter; LVEF, left ventricular ejection fraction; LVMI, left ventricular mass index; LVPWT, left ventricular posterior wall thickness; RWT, relative wall thickness; TRV, tricuspid regurgitation peak velocity.

### LA structure parameters on follow-up

**Figure 2:**
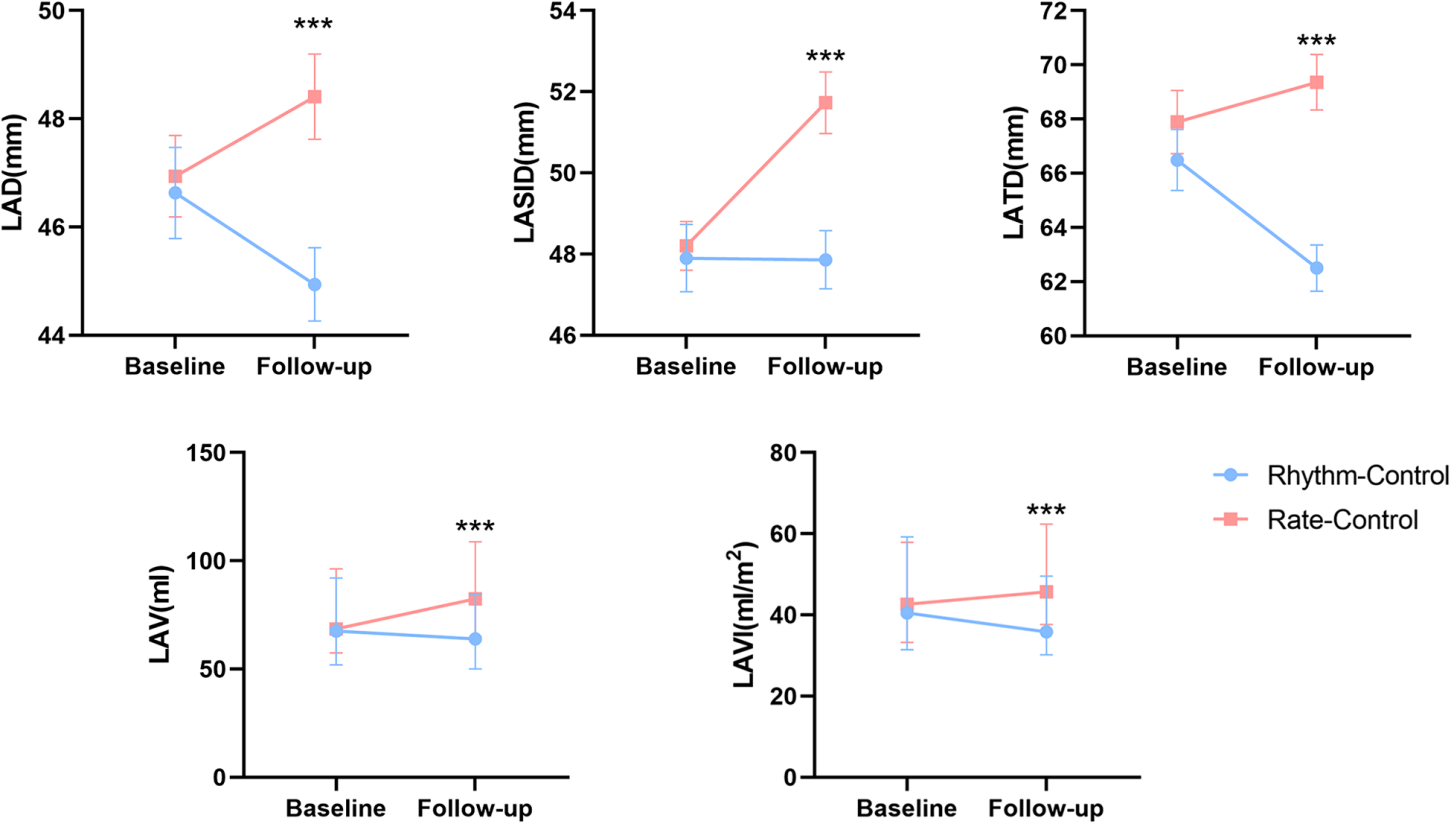
Changes in left atrial structure parameters between baseline and follow-up in rhythm and rate-control groups after PS-matching.

**Figure 3:**
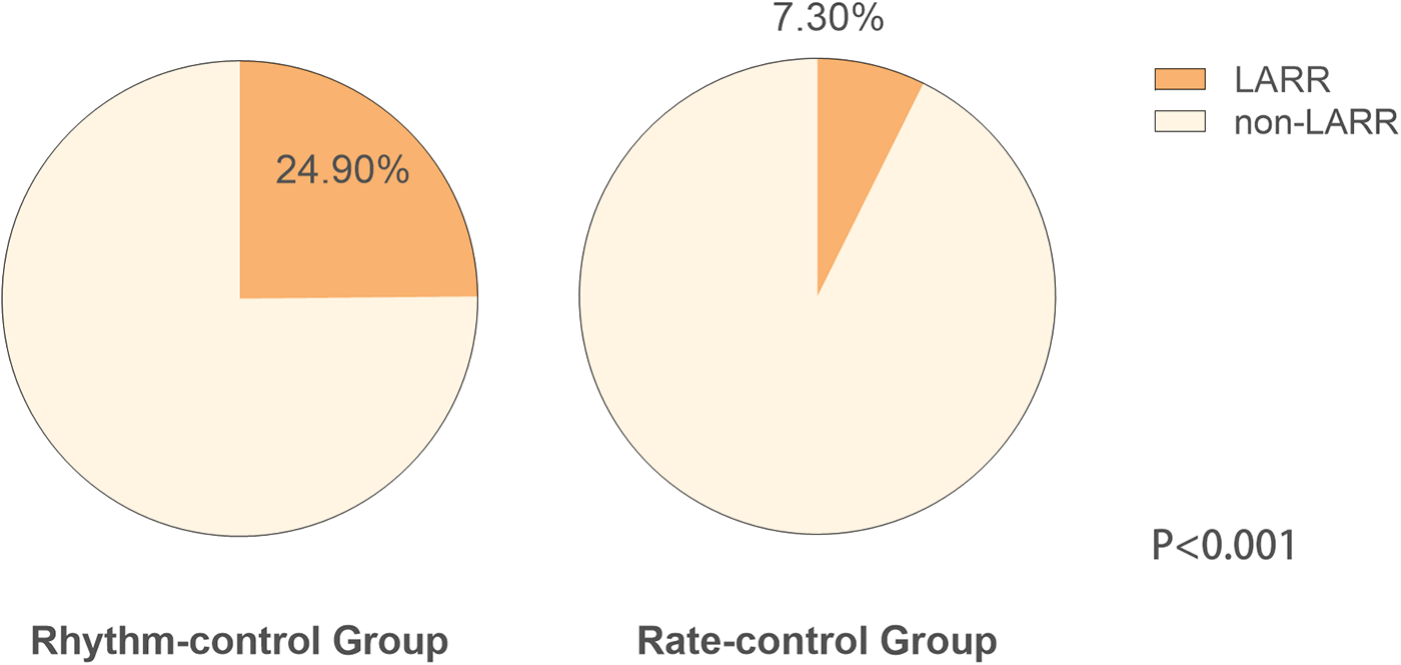
Left atrial reverse remodeling in the rhythm and rate-control groups after PS-matching.

Differences in follow-up echocardiographic parameters were compared between the two groups (Table 2). Compared with the rate-control group, the rhythm-control group showed more significant reductions in LA diameter (LAD) [rhythm control: from 45.88 ± 0.73 to 44.94 ± 0.68 vs. rate control: from 46.65 ± 0.69 to 48.41 ± 0.79, *p* = 0.001], LA superior-inferior diameter (LASID) [rhythm control: from 47.90 ± 0.83 to 47.86 ± 0.72 vs. rate control: from 48.20 ± 0.60 to 51.73 ± 0.76, *p* < 0.001], LA transverse diameter (LATD) [rhythm control: from 66.49 ± 0.97 to 62.50 ± 0.84 vs. rate control: from 66.72 ± 1.03 to 69.24 ± 0.99, *p* < 0.001], LAV [rhythm control: from 67.54 (51.88, 92.12) to 63.95 (50.07, 84.68) vs. rate control: from 68.61 (57.40, 96.28) to 82.44 (64.81, 108.86), *p* < 0.01] and LAVI [rhythm control: from 40.53 (31.43, 59.23) to 35.86 (30.18, 49.59) vs. rate control: from 42.66 (33.25, 57.89) to 45.68 (37.66, 62.34), *p* < 0.01] (Figure 2). Moreover, rhythm-control therapy was also associated with a higher incidence of LA reverse remodeling than traditional rate-control therapy [44 (24.90%) vs. 13 (7.30%), *p* < 0.001] (Figure 3). Other indicators, such as IVS, LVPWT, LVEDD, LVMI, E/e', TRV, and RWT, were similar between the two groups at follow-up.

**Table 2:** Echocardiographic parameters between rhythm and rate-control groups after PS-matching on follow-up.

	Rhythm-control group	Rate-control group	*p* value
	(*N* = 177)	(*N* = 177)	
LVEF (%)	55.34 ± 0.37	53.98 ± 0.47	0.024
LAD (mm)	44.94 ± 0.68	48.41 ± 0.79	0.001
LASID (mm)	47.86 ± 0.72	51.73 ± 0.76	<0.001
LATD (mm)	62.50 ± 0.84	69.24 ± 0.99	<0.001
LAV (ml)	63.95 (50.07, 84.68)	82.44 (64.81, 108.86)	<0.001
LAVI (ml/m^2^)	35.86 (30.18, 49.59)	45.68 (37.66, 62.34)	<0.001
IVS (mm)	11.17 ± 0.23	10.69 ± 0.17	0.094
LVPWT (mm)	10.22 ± 0.10	10.00 ± 0.10	0.121
LVEDD (mm)	47.65 ± 0.43	48.71 ± 0.52	0.117
LVMI (g/m^2^)	102.19 (83.61, 125.83)	97.48 (83.21, 127.02)	0.670
RWT (mm)	0.43 (0.40, 0.46)	0.42 (0.37, 0.45)	0.094
TRV (m/s)	2.65 (2.40, 2.90)	2.70 (2.40, 3.10)	0.170
E/e’	11.4 (8.20, 16.55)	10.5 (8.25, 13.45)	0.250

IVS, interventricular septal thickness; LAD, left atrial diameter; LASID, left atrium superior-inferior diameter; LATD, left atrium transverse diameter; LAV, left atrial volume; LAVI, left atrial volume index; LVEDD, left ventricular end-diastolic diameter; LVEF, left ventricular ejection fraction; LVMI, left ventricular mass index; LVPWT, left ventricular posterior wall thickness; RWT, relative wall thickness; TRV, tricuspid regurgitation peak velocity.

### Adverse outcomes on follow-up

**Figure 4:**
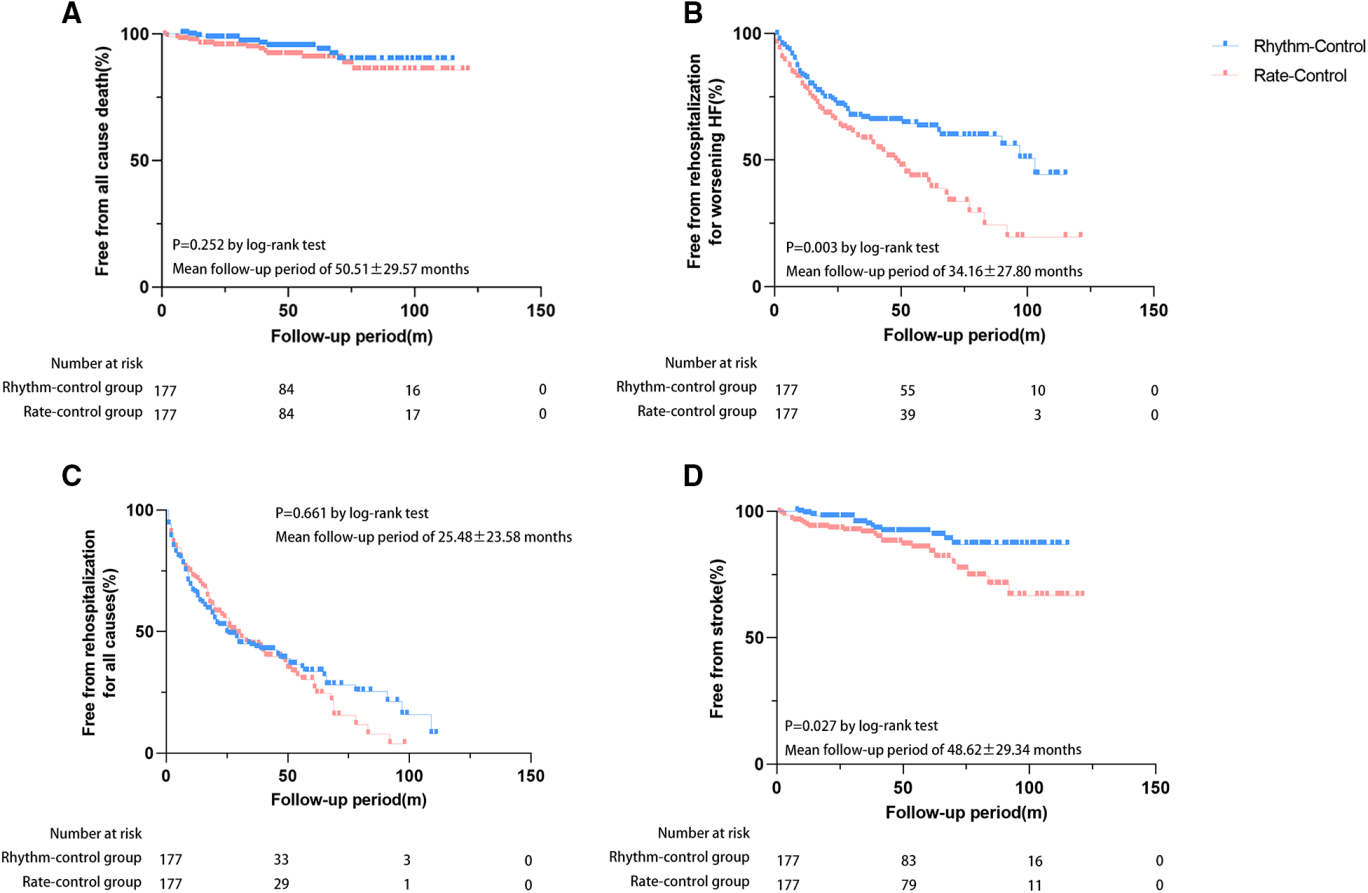
Risks of adverse events between rhythm and rate-control groups after PS-matching. Event-free proportions from (**A**) all-cause mortality, (**B**) HF-related rehospitalization, (**C**) rehospitalization for any cause, (**D**) stroke.

During follow-up, 25 patients died [rate-control: *n* = 15 (8.5%) vs. rhythm-control: *n* = 10 (5.6%)], and 42 cases developed stroke [rate-control: *n* = 27 (15.3%) vs. rhythm-control: *n* = 15 (8.5%)]. A total of 223 patients were rehospitalized [rate-control: *n* = 111 (62.7%) vs. rhythm-control: *n* = 112 (63.3%)], of whom 154 were readmitted for worsening HF [rate-control: *n* = 88 (45.8%) vs. rhythm-control: *n* = 66 (37.3%)]. Kaplan-Meier analysis showed that patients in the rhythm-control group had lower incidences of HF-related rehospitalization (*p* = 0.003) and stroke (*p* = 0.027) when compared to those in the rate-control group (Figure 4). Cox regression analysis also demonstrated that patients treated with rhythm-control therapy experienced lower risks of HF-related rehospitalization after adjusting for three potential confounders (adjusted HR 0.605, 95% CI 0.413–0.887, *p* = 0.010) but not with stroke (adjusted HR 0.589, 95% CI 0.279–1.242, *p* = 0.164) (Table 3). Differences in the risks of hospitalization for any cause and all-cause mortality among the two groups were insignificant either before or after adjustment (Table 3). Survival analysis was also performed to compare the efficacy of different rhythm control strategies for AF and HFpEF, aiming to identify a convenient and inexpensive approach for individuals unsuitable for CA. Surprisingly, CA did not show a remarkable superiority in reducing the risks of preset endpoints over ECV (data not shown).

**Table 3:** Risks of adverse events between rhythm and rate-control groups after PS-matching.

Adverse outcomes	Unadjusted HR (95% CI)	*p* value	Adjusted HR (95% CI)	*p* value
All-cause mortality	0.629 (0.283–1.401)	0.257	0.644 (0.252–1.643)	0.357
HF rehospitalization	0.619 (0.448–0.853)	0.003	0.605 (0.413–0.887)	0.010
Rehospitalization for any cause	0.943 (0.724–1.229)	0.665	0.980 (0.712–1.348)	0.901
Stroke	0.498 (0.265–0.937)	0.031	0.589 (0.279–1.242)	0.164

HR, hazard ratio; CI, confidence interval; HR and CI were derived from Cox proportional hazard models.

Adjusted for β-blocker and digoxin.

## Discussion

Among HFpEF and AF patients, rhythm-control treatment effectively reduced the risk of adverse endpoint. It significantly contributed to improved LA size, characterized by decreased LA volume and a higher rate of LA reverse remodeling compared to rate-control treatment.

Some extensive randomized controlled studies, such as CASTLE-AF, CABANA-HF, and EAST-AFNET4 trials, established that CA therapy for AF and HF, especially HFrEF, was superior to conventional ventricular rate medications in enhancing cardiac functions, improving the quality of life, and reducing the risks of death and admission (7, 8, 10, 12). Based on this, the latest guideline recommends that patients with deteriorating HF and symptomatic AF are encouraged to choose CA on the premise of comprehensive upstream therapy and stroke prevention (15). However, multiple pieces of evidence have shown that HFpEF significantly differs from HFrEF in pathogenesis, clinical manifestations, and prognosis (16–18). Patients with HFpEF are more female, have lower NP levels, and show a higher prevalence of atrial fibrillation and hypertension but a lower incidence of ischemic heart disease (16). Several large RCT trials have reported that, unlike HFrEF, most HFpEF patients respond poorly to conventional HF medical therapy (ACEI/ARB/ARNI, β-blocker, MRA) apart from loop diuretics and sodium-dependent glucose transporters-2 inhibitors (19–22). Recent basic research found that pyroptosis of adipocytes from epicardial adipose tissue was deeply involved in the myocardial damage in a mouse model of HFpEF, (23) implying the great importance of non-cardiac mechanisms in HFpEF. Therefore, there should also be more evidence of the clinical benefits of CA in HFpEF. Black-Maier et al. reported that CA significantly improved NYHA class and alleviated the symptoms in HFpEF and AF patients (24). A sizeable retrospective study revealed that compared with the rate control therapy, rhythm control was more effective in decreasing all-cause mortality in elderly AF and HFpEF patients after 1-year follow-up (25). Another study involving 85 HFpEF and AF patients showed that CA markedly reduced the rehospitalization risk for HF (26). Two meta-analyses showed that CA reduced the rehospitalization rate for HF and all-cause mortality in HFpEF and AF patients (27, 28). Our findings were consistent with these studies, further elucidating the clinical benefits of rhythm control in decreasing the incidence of HF-related rehospitalization for HFpEF and AF. Two other studies reported that CA decreased the risk of ischemic stroke [HR 0.69, 95% CI (0.51–0.93) and RR 0.22; CI (0.09–0.56)] (29, 30). Our study also confirmed that rhythm treatment was associated with a lower stroke hazard, even if there was no statistical difference after adjustment. However, no significant reduction in the risks of all-cause mortality and rehospitalization was observed for rhythm treatment in the study. This may be due to the small sample size, lower mortality rate, and relatively shorter follow-up period. Thus, large clinical trials are necessary to be carried out to detect the impact of rhythm treatments in these patients.

HFpEF and AF are closely associated with atrial failure, defined as any atrial dysfunction resulting in impaired HF-related symptoms and worsening life expectancy in the absence of significant valvular or ventricular abnormalities, which was proposed in 2020 (31). LA structure remodeling, manifested by LA dilation, is one of the most critical underlying mechanisms of atrial failure, leading to increased incidences of HFpEF and AF (4, 32). In turn, AF induces oxidative stress, atrial enlargement, calcium overload, inflammation, immune cell infiltration, microRNA expression, and myofibroblast activation, resulting in atrial remodeling, increasing the risk of HF and, ultimately, a vicious cycle (33–36). Thus, timely and effective intervention of AF may promote LA reverse remodeling and improve clinical endpoints. In this study, we analyzed some LA indicators and found that they were significantly decreased after rhythm-control therapy, with a higher proportion of LARR. These findings showed that rhythm treatment had a more favorable effect on reversing LA structure remodeling. Rhythm treatment may improve LA structure remodeling, greatly stabilizing hemodynamics and preventing HF deterioration.

In addition to CA, left atrial appendage closure (LAAC) is an effective strategy to reduce the risk of ischemic stroke/TIA/systemic embolism during follow-up (37). The combination of CA and LAAC has been proven beneficial to the long-term prognosis of patients with AF. A meta-analysis demonstrated that ablation with the vein of Marshall ethanol infusion (VOM-ABL) was associated with higher rates of successful mitral isthmus (MI) block and long-term freedom from AF/AT and was comparable safety compared with ablation alone in patients with AF followed over 1 year or more (38). Moreover, a significant proportion of patients cannot receive CA treatment due to various reasons (old age, over-dilated LA, multiple comorbidities, and worse cardiac functions) in clinical practice. Therefore, it is necessary to identify an alternative treatment strategy for this population. Interestingly, patients who received ECV and those subjected to CA exhibited comparable benefits in reducing the incidence of adverse events, indicating that ECV may be an effective alternative for those who cannot tolerate CA or may benefit less. These recent advancements also needed to be verified in the HFpEF population through large multi-center trials.

We admitted that there were several limitations involved in this study. Firstly, the study was neither a randomized controlled nor a large multi-center. Thus, our findings may not apply to all populations. Secondly, all participants were retrospectively identified; selection bias was inevitable. Thirdly, as there was no significant difference in the number of cases between the two groups, conducting a successful 1:1 match based on all potential confounders is unavailable, resulting in some difference in baseline data among the two groups. Fourthly, owing to financial factors and patients' wishes, implanting the cardiac monitor to record EKG for all patients may be difficult. Thus, some recurrent atrial arrhythmia might be missed. Lastly, the endpoints were mainly obtained by telephone interview, which may cause bias.

In conclusion, rhythm-control therapy reverses LA structure remodeling and is associated with improved clinical outcomes; therefore, it is an optimal therapeutic option for AF in HFpEF patients.

## Data Availability

The original contributions presented in the study are included in the article/Supplementary Material, further inquiries can be directed to the corresponding authors.

## References

[1] Savarese G Becher PM Lund LH Seferovic P Rosano GMC Coats A. Global burden of heart failure: a comprehensive and updated review of epidemiology. Cardiovasc Res*.* (2023) 118(17):3272–87. 10.1093/cvr/cvac013 35150240

[2] Zakeri R Chamberlain AM Roger VL Redfield MM. Temporal relationship and prognostic significance of atrial fibrillation in heart failure patients with preserved ejection fraction: a community-based study. Circulation*.* (2013) 128(10):1085–93. 10.1161/circulationaha.113.001475 23908348 PMC3910441

[3] Vermond RA Geelhoed B Verweij N Tieleman RG Van der Harst P Hillege HL Incidence of atrial fibrillation and relationship with cardiovascular events, heart failure, and mortality: a community-based study from The Netherlands. J Am Coll Cardiol*.* (2015) 66(9):1000–7. 10.1016/j.jacc.2015.06.1314 26314526

[4] Kotecha D Lam CS Van Veldhuisen DJ Van Gelder IC Voors AA Rienstra M. Heart failure with preserved ejection fraction and atrial fibrillation: vicious twins. J Am Coll Cardiol*.* (2016) 68(20):2217–28. 10.1016/j.jacc.2016.08.048 27855811

[5] Heidenreich PA Bozkurt B Aguilar D Allen LA Byun JJ Colvin MM 2022 AHA/ACC/HFSA guideline for the management of heart failure: a report of the American college of cardiology/American heart association joint committee on clinical practice guidelines. Circulation*.* (2022) 145(18):e895–1032. 10.1161/cir.0000000000001063 35363499

[6] McDonagh TA Metra M Adamo M Gardner RS Baumbach A Böhm M 2021 ESC guidelines for the diagnosis and treatment of acute and chronic heart failure. Eur Heart J*.* (2021) 42(36):3599–726. 10.1093/eurheartj/ehab368 34447992

[7] Marrouche NF Brachmann J Andresen D Siebels J Boersma L Jordaens L Catheter ablation for atrial fibrillation with heart failure. N Engl J Med*.* (2018) 378(5):417–27. 10.1056/NEJMoa1707855 29385358

[8] Hunter RJ Berriman TJ Diab I Kamdar R Richmond L Baker V A randomized controlled trial of catheter ablation versus medical treatment of atrial fibrillation in heart failure (the CAMTAF trial). Circ Arrhythm Electrophysiol*.* (2014) 7 (1):31–8. 10.1161/circep.113.000806 24382410

[9] Jones DG Haldar SK Hussain W Sharma R Francis DP Rahman-Haley SL A randomized trial to assess catheter ablation versus rate control in the management of persistent atrial fibrillation in heart failure. J Am Coll Cardiol*.* (2013) 61(18):1894–903. 10.1016/j.jacc.2013.01.069 23500267

[10] Rillig A Magnussen C Ozga AK Suling A Brandes A Breithardt G Early rhythm control therapy in patients with atrial fibrillation and heart failure. Circulation*.* (2021) 144 (11):845–58. 10.1161/circulationaha.121.056323 34328366 PMC8456351

[11] Prabhu S Taylor AJ Costello BT Kaye DM McLellan AJA Voskoboinik A Catheter ablation versus medical rate control in atrial fibrillation and systolic dysfunction: the CAMERA-MRI study. J Am Coll Cardiol*.* (2017) 70 (16):1949–61. 10.1016/j.jacc.2017.08.041 28855115

[12] Packer DL Piccini JP Monahan KH Al-Khalidi HR Silverstein AP Noseworthy PA Ablation versus drug therapy for atrial fibrillation in heart failure: results from the CABANA trial. Circulation*.* (2021) 143 (14):1377–90. 10.1161/circulationaha.120.050991 33554614 PMC8030730

[13] Pieske B Tschöpe C de Boer RA Fraser AG Anker SD Donal E How to diagnose heart failure with preserved ejection fraction: the HFA-PEFF diagnostic algorithm: a consensus recommendation from the heart failure association (HFA) of the European society of cardiology (ESC). Eur Heart J*.* (2019) 40(40):3297–317. 10.1093/eurheartj/ehz641 31504452

[14] Tops LF Delgado V Bertini M Marsan NA Den Uijl DW Trines SA Left atrial strain predicts reverse remodeling after catheter ablation for atrial fibrillation. J Am Coll Cardiol*.* (2011) 57(3):324–31. 10.1016/j.jacc.2010.05.063 21232671

[15] Hindricks G Potpara T Dagres N Arbelo E Bax JJ Blomström-Lundqvist C 2020 ESC guidelines for the diagnosis and management of atrial fibrillation developed in collaboration with the European association for cardio-thoracic surgery (EACTS): the task force for the diagnosis and management of atrial fibrillation of the European society of cardiology (ESC) developed with the special contribution of the European heart rhythm association (EHRA) of the ESC. Eur Heart J*.* (2021) 42(5):373–498. 10.1093/eurheartj/ehaa612 32860505

[16] Gevaert AB Kataria R Zannad F Sauer AJ Damman K Sharma K Heart failure with preserved ejection fraction: recent concepts in diagnosis, mechanisms and management. Heart*.* (2022) 108(17):1342–50. 10.1136/heartjnl-2021-319605 35022210

[17] Drazner MH. Insights from the history and physical examination in HFpEF or HFrEF: similarities and differences. JACC Heart Fail. (2021) 9(5):398–400. 10.1016/j.jchf.2021.02.009 33926731

[18] Li Z Shi Y Xia Y Wu L Li H Zhou R Disparate clinical characteristics and prognosis of HFpEF versus HFrEF phenotype of diabetic cardiomyopathy. J Clin Med*.* (2023) 12(4):1565. 10.3390/jcm12041565 36836101 PMC9960597

[19] Solomon SD McMurray JJV Anand IS Ge J Lam CSP Maggioni AP Angiotensin-Neprilysin inhibition in heart failure with preserved ejection fraction. N Engl J Med*.* (2019) 381(17):1609–20. 10.1056/NEJMoa1908655 31475794

[20] Cleland JGF Bunting KV Flather MD Altman DG Holmes J Coats AJS Beta-blockers for heart failure with reduced, mid-range, and preserved ejection fraction: an individual patient-level analysis of double-blind randomized trials. Eur Heart J*.* (2018) 39(1):26–35. 10.1093/eurheartj/ehx564 29040525 PMC5837435

[21] Pitt B Pfeffer MA Assmann SF Boineau R Anand IS Claggett B Spironolactone for heart failure with preserved ejection fraction. N Engl J Med*.* (2014) 370(15):1383–92. 10.1056/NEJMoa1313731 24716680

[22] Vaduganathan M Docherty KF Claggett BL Jhund PS de Boer RA Hernandez AF SGLT-2 inhibitors in patients with heart failure: a comprehensive meta-analysis of five randomised controlled trials. Lancet*.* (2022) 400(10354):757–67. 10.1016/s0140-6736(22)01429-5 36041474

[23] Xia YY Shi Y Li Z Li H Wu LD Zhou WY Involvement of pyroptosis pathway in epicardial adipose tissue—myocardium axis in experimental heart failure with preserved ejection fraction. Biochem Biophys Res Commun*.* (2022) 636(Pt 2):62–70. 10.1016/j.bbrc.2022.10.109 36356543

[24] Black-Maier E Ren X Steinberg BA Green CL Barnett AS Rosa NS Catheter ablation of atrial fibrillation in patients with heart failure and preserved ejection fraction. Heart Rhythm*.* (2018) 15(5):651–7. 10.1016/j.hrthm.2017.12.001 29222043

[25] Kelly JP DeVore AD Wu JJ Hammill BG Sharma A Cooper LB Rhythm control versus rate control in patients with atrial fibrillation and heart failure with preserved ejection fraction: insights from get with the guidelines-heart failure. J Am Heart Assoc*.* (2019) 8(24):e011560. 10.1161/JAHA.118.011560 31818219 PMC6951063

[26] Fukui A Tanino T Yamaguchi T Hirota K Saito S Okada N Catheter ablation of atrial fibrillation reduces heart failure rehospitalization in patients with heart failure with preserved ejection fraction. J Cardiovasc Electrophysiol. (2020) 31(3):682–8. 10.1111/jce.14369 31985099

[27] Gu G Wu J Gao X Liu M Jin C Xu Y. Catheter ablation of atrial fibrillation in patients with heart failure and preserved ejection fraction: a meta-analysis. Clin Cardiol. (2022) 45(7):786–93. 10.1002/clc.23841 35544952 PMC9286329

[28] Androulakis E Sohrabi C Briasoulis A Bakogiannis C Saberwal B Siasos G Catheter ablation for atrial fibrillation in patients with heart failure with preserved ejection fraction: a systematic review and meta-analysis. J Clin Med*.* (2022) 11(2):288. 10.3390/jcm11020288 35053984 PMC8779551

[29] Friberg L Tabrizi F Englund A. Catheter ablation for atrial fibrillation is associated with lower incidence of stroke and death: data from Swedish health registries. Eur Heart J*.* (2016) 37(31):2478–87. 10.1093/eurheartj/ehw087 26984861

[30] Rahman A Hasani A Moussa O Kumar S Jahufar F Saeed O Efficacy of catheter ablation of atrial fibrillation in heart failure with preserved ejection fraction. J Card Fail*.* (2019) 25(8):S84–5. 10.1016/j.cardfail.2019.07.241

[31] Bisbal F Baranchuk A Braunwald E Bayés de Luna A Bayés-Genís A. Atrial failure as a clinical entity: JACC review topic of the week. J Am Coll Cardiol*.* (2020) 75(2):222–32. 10.1016/j.jacc.2019.11.013 31948652

[32] Reddy YNV Obokata M Verbrugge FH Lin G Borlaug BA. Atrial dysfunction in patients with heart failure with preserved ejection fraction and atrial fibrillation. J Am Coll Cardiol*.* (2020) 76(9):1051–64. 10.1016/j.jacc.2020.07.009 32854840 PMC7455760

[33] Jalife J Kaur K. Atrial remodeling, fibrosis, and atrial fibrillation. Trends Cardiovasc Med*.* (2015) 25(6):475–84. 10.1016/j.tcm.2014.12.015 25661032 PMC5658790

[34] Gómez-Outes A Lagunar-Ruíz J Terleira-Fernández AI Calvo-Rojas G Suárez-Gea ML Vargas-Castrillón E. Causes of death in anticoagulated patients with atrial fibrillation. J Am Coll Cardiol*.* (2016) 68(23):2508–21. 10.1016/j.jacc.2016.09.944 27931607

[35] Lam CS Rienstra M Tay WT Liu LC Hummel YM van der Meer P Atrial fibrillation in heart failure with preserved ejection fraction: association with exercise capacity, left ventricular filling pressures, natriuretic peptides, and left atrial volume. JACC Heart Fail. (2017) 5(2):92–8. 10.1016/j.jchf.2016.10.005 28017355

[36] Wu LD Li F Chen JY Zhang J Qian LL Wang RX. Analysis of potential genetic biomarkers using machine learning methods and immune infiltration regulatory mechanisms underlying atrial fibrillation. BMC Med Genomics*.* (2022) 15(1):64. 10.1186/s12920-022-01212-0 35305619 PMC8934464

[37] Li F Sun JY Wu LD Hao JF Wang RX. The long-term efficacy and safety of combining ablation and left atrial appendage closure: a systematic review and meta-analysis. J Cardiovasc Electrophysiol*.* (2021) 32(11):3068–81. 10.1111/jce.15230 34453379

[38] Li F Sun JY Wu LD Zhang L Qu Q Wang C The long-term outcomes of ablation with vein of marshall ethanol infusion vs. ablation alone in patients with atrial fibrillation: a meta-analysis. Front Cardiovasc Med*.* (2022) 9:871654. 10.3389/fcvm.2022.871654 35571170 PMC9098965

